# Dynamic Remodeling of Plant Cytoskeleton in Response to Environmental Stress

**DOI:** 10.3390/biology15100752

**Published:** 2026-05-09

**Authors:** Piaojuan Chen, Zichun Xia, Huicong Wu, Jiayang Zhang, Yadan Liu, Qin Wang, Ming Zhong

**Affiliations:** College of Life Science, Basic Forestry and Proteomics Research Center, Fujian Agriculture and Forestry University, Fuzhou 350002, China

**Keywords:** cytoskeleton, dynamic remodeling, microtubule-associated proteins (MAPs), actin-binding proteins (ABPs), environmental stress

## Abstract

Enhancing crop stress tolerance to ensure global food security is one of the core challenges in agricultural science. Plants predominantly face biotic and abiotic stresses, to which they respond by activating finely regulated signal perception and transduction pathways, thereby improving their survival in adverse environments. The plant cytoskeleton, composed of microtubules and actin filaments, plays a pivotal role in this adaptive process. It functions both as a hub for integrating external stress signals and as a key regulator of downstream signaling and cellular responses. Upon stress, the cytoskeleton undergoes dynamic remodeling, a process driven mainly by microtubule-associated proteins (MAPs) and actin-binding proteins (ABPs). This review systematically summarizes current knowledge on cytoskeletal remodeling in plants under environmental stress, particularly focusing on the functions and mechanisms of MAPs and ABPs in cytoskeletal remodeling. Furthermore, it outlines the regulatory network through which the plant cytoskeleton orchestrates stress adaptation.

## Simple Summary

Enhancing crop stress tolerance is crucial for global food security. Plants encounter both biotic and abiotic stresses and respond through finely tuned signal perception and transduction pathways. The plant cytoskeleton, comprising microtubules and actin filaments, serves as a central hub for integrating external stress signals and regulating downstream cellular responses. We systematically summarize the cytoskeletal rearrangement processes in plant cells induced by abiotic and biotic stresses, such as biotic stress and abiotic stresses (temperature, drought, salinity and light), with a particular focus on the functions and molecular mechanisms of MAPs and ABPs. This work outlines the molecular regulatory network by which the cytoskeleton mediates plant stress adaptation, and provides theoretical guidance and practical implications for the screening of stress-tolerant crop cultivars.

## 1. Introduction

The cytoskeleton is involved in the numerous cellular signals in response to environmental stresses. Such stresses, including both biotic threats, such as pathogen attack [1,2], and abiotic stresses, such as drought [3], temperature extremes [4,5,6] and light exposure [7,8], not only severely compromise plant growth, crop quality, and yield but can also directly threaten plant survival. To mitigate these threats, plants have evolved sophisticated defense mechanisms, among which the cytoskeletal system has emerged as a pivotal signaling and structural network for environmental signal perception and adaptation.

As a dynamic and versatile scaffold network ubiquitously distributed in eukaryotic cells, the cytoskeleton is fundamental to numerous cellular processes, from determining cell shape and polarity to facilitating intracellular transport and division [9]. Beyond these housekeeping functions, it plays a central role in integrating stress signal transduction. Acting as an early stress sensor, the cytoskeleton detects external physical, chemical, and biological cues and translates them into coordinated intracellular responses [10,11]. Indeed, the plant cytoskeleton is not merely a passive detector but an active participant in signaling cascades. It responds to diverse developmental and environmental signals through precise remodeling of its own architecture and dynamics [10,12], thereby orchestrating physiological outcomes against specific environmental stressors.

The plant cytoskeleton primarily consists of two dynamic and interconnected filament systems: microtubules (MTs), polymers of α/β-tubulin heterodimers, and actin filaments (AFs), polymers of globular actin. As part of the broader eukaryotic cytoskeleton, this network forms a contiguous system within all cells, where both MTs and AFs are associated with numerous cellular processes [13,14,15,16,17]. In short, MTs are hypothesized to play a role in maintaining cell polarity, whereas AFs ensure the targeted delivery of vesicles that carry plasma membrane and cell-wall components to the site of growth [18]. Additionally, AFs support the formation of penetration barriers by recruiting defense-related products to the subcellular site of fungal attack [19]. As a putative mechanism underpinning MTs and AFs-associated cell signaling, work by Trozzi and Kunkowska et al. [20] and Luo et al. [21] noted that MT form a cortical “fence,” restricting protein diffusion and organizing receptors to maintain polarity. AFs, in contrast, power clathrin-mediated endocytosis, driving vesicle formation and cargo transport. Notably, microtubules and actin filaments coordinate their responses to environmental changes through distinct yet interconnected cell-signaling pathways [22,23]. This review aims to summarize the mechanisms of plant cytoskeleton remodeling under biotic and abiotic stresses, particularly the functions and mechanisms of cytoskeleton-binding proteins, including microtubule-associated proteins (MAPs) and actin-binding proteins (ABPs), in response to the environment.

## 2. Cytoskeletal Dynamics

**Figure 1:**
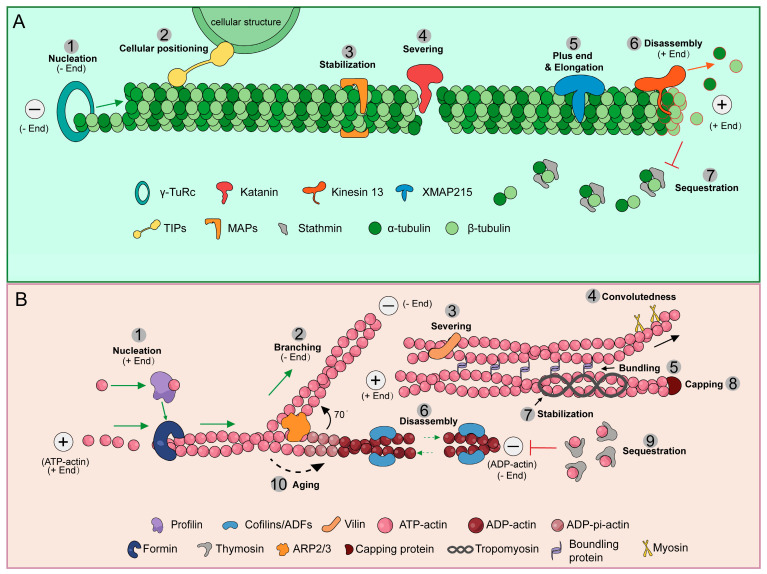
Cytoskeleton dynamics mediated by MAPs and ABPs. (**A**). MAPs regulate microtubule dynamics. ① γ-TuRC initiates de novo microtubule assembly [28]. ② +TIPs specifically bind to microtubule minus ends to mediate cellular interactions of microtubule and cellular structure to direct organelle positioning [29]. ③ Classical MAPs, such as Tau/MAP2, stabilize microtubules and promote bundling [30]. ④ Katanin severs microtubules, increasing the number of available microtubule ends [31]. ⑤ XMAP215 accelerates plus-end elongation [32]. ⑥ Kinesin-13 depolymerizes microtubules by curling their terminal protofilaments, leading to disassembly [33]. ⑦ Stathmin drives microtubule depolymerization mainly by tubulin dimer sequestration [34]. (**B**). ABPs regulate actin filament dynamics. ① Profilin delivers G-actin to filament barbed ends to promote polymerization [35]. ② Arp2/3 nucleates branched actin networks by binding to the sides of existing filaments [36]. ③ Villin severs actin filaments by binding to their barbed ends [37]. ④ Myosin as a molecular motor that uses ATP hydrolysis to generate force for contraction or transportation [38]. ⑤ Bundling proteins crosslink actin filaments into parallel, organized bundles [39,40]. ⑥ Cofilins/ADFs sever filaments and enhance ADP-actin dissociation from pointed ends [41]. ⑦ Tropomyosin stabilizes actin filaments by binding along their length [42]. ⑧ Capping proteins stabilize filament by binding to barbed ends to block actin subunit addition or loss [43]. ⑨ Thymosin sequesters G-actin to prevent spontaneous polymerization [44]. ⑩ “Aging” process: the biochemical transition of actin subunits from an ATP-bound to an ADP-bound state.

The plant cytoskeleton, composed of actin filaments (AFs) and microtubules (MTs), is not a static scaffold but a highly dynamic structure. Its precise and rapid remodeling, referred to as cytoskeleton dynamics, is crucial for plant adaptation and survival. Actin-binding proteins (ABPs) and microtubule-associated proteins (MAPs) play a major role in plant cytoskeleton remodeling, regulating cytoskeleton nucleation, elongation, breakage, and cross-linking [24,25,26,27]. Although some of these proteins (Figure 1) have been largely characterized in animal systems, they still provide a foundational framework for understanding how the dynamic plasticity of the plant microtubule cytoskeleton is achieved in response to environmental changes. ABPs and MAPs can be considered the regulatory hubs of the cytoskeleton, through which cells translate internal and external signals into adaptive cytoskeleton remodeling, thereby regulating plant growth.

### 2.1. MAPs Involved in Microtubule Dynamics Regulation

In cells, microtubule dynamics are precisely regulated by a series of specialized microtubule-associated proteins (Figure 1A). This regulatory cascade begins with γ-Tubulin Ring Complex (γ-TuRC)-dependent microtubule nucleation, where γ-TuRC acts as a template to catalyze the formation of new microtubules that branch from existing filaments [28]. The growing plus ends of microtubules are specifically recognized and bound by Plus-End Tracking Proteins (+TIPs), which modulate microtubule dynamics and mediate interactions with the cell cortex or organelles to guide directional growth [29]. Along the microtubule lattice, classical microtubule-associated proteins, such as Tau and Microtubule-Associated Protein 2 (MAP2), bind to the filaments to stabilize them and regulate inter-microtubule spacing and bundling [30]. Microtubule length and distribution are tightly controlled by the severing protein Katanin, which hydrolyzes ATP to cut microtubules, thereby increasing the number of microtubule ends and remodeling the microtubule network architecture [31]. While the microtubule polymerase Xenopus Microtubule-Associated Protein of 215 kDa (XMAP215) promotes rapid elongation of the positive microtubule tip by continuously adding tubulin dimers [32]. To maintain dynamic homeostasis, the depolymerization kinesin-13 actively promotes microtubule depolymerization by inducing protofilament coiling at the microtubule tips [33]. Simultaneously, the microtubule-destabilizing protein stathmin (Oncoprotein 18: Op18) inhibits polymerization and promotes depolymerization by sequestering free tubulin dimers or directly binds to and catalyzes the depolymerization of microtubule ends, thereby reducing the pool of subunits available for assembly [34]. These regulatory mechanisms, largely characterized in animal systems, provide a foundational framework for understanding how the dynamic plasticity of the plant microtubule cytoskeleton is achieved in response to environmental changes.

### 2.2. ABPs Involved in Actin Filament Dynamic Regulation

The dynamic of the actin cytoskeleton is also coordinated by a variety of regulatory proteins (Figure 1B). Nucleation is initiated by specific factors, primarily the actin-related protein 2/3 (Arp2/3) complex and formins. The Arp2/3 complex is activated at the sides of existing filaments to nucleate new filaments at a characteristic 70° angle, thereby generating dense, branched networks that produce mechanical force for cell edge protrusion [36]. Once nucleated, filament elongation is facilitated by profilin, which binds to G-actin monomers and delivers them to the growing barbed end [35]. Formins initiate the *de novo* assembly of linear actin filaments. They remain processively bound to the growing barbed end, protecting it from capping proteins and enabling the rapid elongation of long, unbranched filaments [45,46,47]. Filament stability and architecture are further modulated by proteins such as Tropomyosin, which binds along filament sides to provide stability [42], and bundling proteins, which crosslink filaments into parallel bundles [39,40]. The precise length and turnover of filaments are controlled by different mechanisms: capping protein binds to barbed ends to terminate growth [43,48], Villin severs filaments [37,49], and Cofilin/ADF promotes depolymerization by severing filaments and accelerating monomer dissociation from pointed ends [41]. Cellular force generation and transport are driven by myosin, a molecular motor that moves along actin filaments by hydrolysing ATP [38]. Finally, the availability of polymerization-competent subunits is regulated by Thymosin, which sequesters G-actin to maintain a soluble monomer pool [44], and by the intrinsic “aging” process whereby actin subunits transition from an ATP-bound to an ADP-bound state, affecting their assembly abilities [50]. Taken together, the dynamics of the actin cytoskeleton are well orchestrated by a variety of regulatory proteins that precisely control the nucleation, elongation, stabilization, severing, and turnover of actin filaments.

## 3. Cytoskeletal Dynamics in Response to Environmental Stress

The plant cytoskeleton not only maintains cell structure and material transportation but also serves as a central hub for responding to multiple environmental stresses. To cope with complex and ever-changing environments, including biotic stresses such as pathogen invasion [1] and abiotic stresses such as drought [3], temperatures stress [4,5], salt stress [51,52,53,54], and light stress [55,56], plants have evolved a sophisticated defense system centered on cytoskeletal remodeling. In the following sections, we will focus on the signal transduction networks of the cytoskeleton in response to biotic and abiotic stresses. MAPs and ABPs involved in environmental stress in plants are listed in Table 1 and Table 2.

### 3.1. Biotic Stresses: Defensive Cytoskeletal Rearrangement Under Pathogen Invasion

#### 3.1.1. Microtubule in Response to Plant Pathogen Invasion

Plant defense signaling against bacterial and fungal pathogens involves at least two major branches of the innate immune system, called microbial- or pathogen-associated molecular patterns (MAMPS or PAMPs)-triggered immunity (PTI) and effector-triggered immunity (ETI) [105]. PTI uses transmembrane pattern recognition receptors (PRRs) that respond to slowly evolving microbial- or pathogen-associated molecular patterns (MAMPS or PAMPs). In turn, pathogens deliver effectors to suppress PTI as a virulence strategy. To counter this virulence strategy, plants have evolved to perceive pathogen effectors, triggering a defense response termed effector-triggered immunity (ETI). ETI results in the activation of robust immune signaling, often characterized by localized cell death (i.e., the hypersensitive response (HR) [106,107].

During plant–pathogen interactions, the cytoskeleton undergoes rapid and dynamic rearrangements, which are essential for orchestrating robust resistance in host plants against microbial invaders [12]. Plant hormones, such as ethylene [108,109], jasmonic acid (JA) [110], and gibberellins (GAs) [111], play essential roles in plant defense against pests and pathogens. During plant–pathogen interactions, the dynamics of gibberellin (GA) levels are critically shaped by the pathogen’s lifestyle [112]. Biotrophic pathogens often manipulate host GA metabolism to elevate GA levels, which promotes the degradation of DELLA proteins and suppresses salicylic acid (SA)-mediated defenses, thereby facilitating infection [113]. In contrast, resistance against necrotrophic pathogens is frequently associated with a rapid decrease in GA content, leading to the stabilization of DELLA proteins that subsequently enhance jasmonic acid/ethylene (JA/ET)-dependent defense responses [113,114].

**Figure 2:**
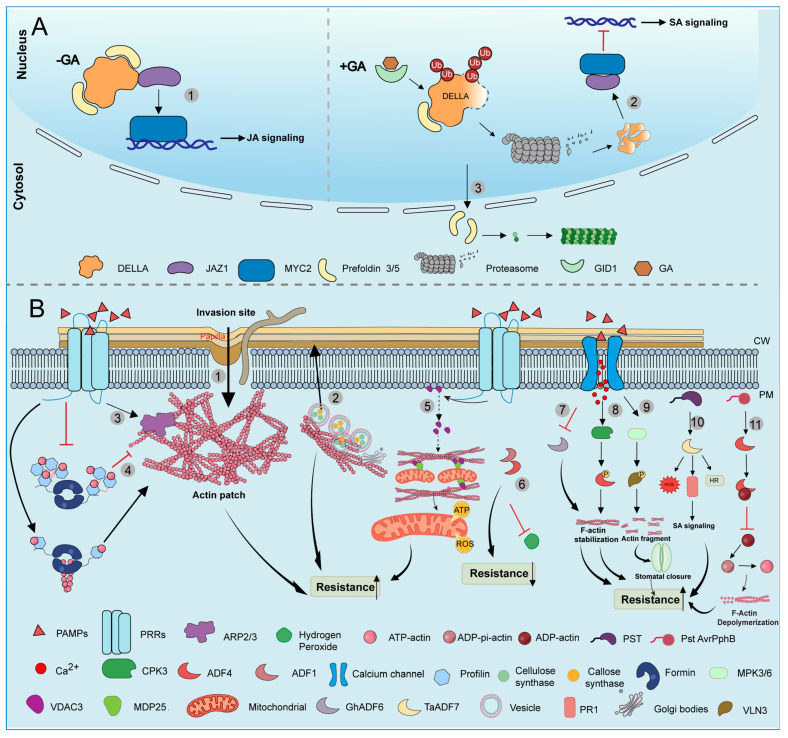
Regulatory network of the cytoskeletal response to biotic stress. (**A**). GA-DELLA pathway response to biotic stress. ① In the absence of GA, DELLA proteins inhibit prefoldin 3/5 and sequester JAZ1, thereby activating MYC2-dependent JA signaling while suppressing the SA pathway [115]. ② GA triggers the GID1-mediated ubiquitination and degradation of DELLA proteins. And then, degradation of DELLA proteins frees JAZ1, which can suppress MYC2, shifting the plant’s focus from defense to growth promotion and cell elongation [115]. ③ DELLA degradation releases prefoldin 3/5 to the cytoplasm, where it facilitates tubulin folding and microtubule reorganization to drive cell elongation [116]. (**B**). Actin filament dynamic response to biotic stress. ① Pathogen-induced actin patches orchestrate a wall-focused defense by directing callose and antimicrobial secretion [117]. ② Plants utilize polarized, cytoskeleton-guided exocytosis to deliver defense materials to infection sites [118]. ③ PAMP recognition activates the WAVE/SCAR-ARP2/3 pathway to nucleate branched actin networks [73,119]. ④ Under normal conditions, AtPRF3 tends to form oligomers, and inhibits formin-mediated actin nucleation. Upon pathogen invasion, PAMP signaling induces the dissociation of AtPRF3 oligomers into monomers, relieving this inhibition and increasing actin filament abundance to resist pathogen invasion [74]. ⑤ PAMP-triggered Ca^2+^ signaling initiates the MDP25-VDAC3 complex, which promotes actin bundling to drive mitochondrial fusion, thereby enhancing ATP/mROS production and immunity [94]. ⑥ Suppressing AtADF1 and AtADF4 confers enhanced resistance to Golovinomyces orontii, by correlating with H_2_O_2_ accumulation and cell death specifically in infected cells [82]. ⑦ GhADF6 strengthens host immunity (ROS and callose deposition) and serves as a critical barrier against the vascular spread of *Verticillium dahlia* [93]. ⑧ Pathogen-induced Ca^2+^ influx activates CPK3 to phosphorylate ADF4, inactivating its depolymerizing function and stabilizing actin filaments [83]. ⑨ MPK3/6 (mitogen-activated protein kinases) phosphorylates VLN3 to remodel actin and activate stomatal immunity, which is critical for bacterial resistance in *Arabidopsis* [96]. ⑩ TaADF7-mediated actin dynamics reinforce wheat defense against *Puccinia striiformis* f. sp. *tritici* by activating ROS-dependent HR [79]. ⑪ Loss of *AtADF4* confers on *Arabidopsis* enhanced susceptibility to *P. syringae* expressing AvrPphB [80].

GA signaling orchestrates a synergistic regulatory network that links microtubule (MT) cytoskeleton remodeling with JA-mediated defense responses, primarily through the central regulatory hub DELLA proteins (Figure 2A). Under normal growth conditions (no/low GA), DELLA proteins accumulate and interact with jasmonate (JA) signaling repressor jasmonate ZIM-domain protein 1 (JAZ1), which relieves JAZ1-mediated inhibition of the transcription factor MYC2 and thus potentiates JA-responsive gene expression. Conversely, elevated GA levels trigger DELLA degradation, which allows JAZ1 to bind and inhibit MYC2, suppressing its transcriptional activity [115]. Alternatively, upon GA perception, the GA–GID1 (Gibberellin-Insensitive Dwarf1) receptor complex triggers the ubiquitination and degradation of DELLA proteins, thereby releasing prefoldin 3/5, which subsequently forms functional hexameric complexes in the cytoplasm to enhance tubulin dimer utilization and indirectly affect the polymerization of microtubules [116]. Thus, the interactions of DELLA with both prefoldin 3/5 and JAZ1 illustrate how a single protein node can synchronize cytoskeletal dynamics with phytohormone signaling to optimize plant adaptation during biotic stress (Figure 2A).

#### 3.1.2. Microfilament Response to Plant Pathogen Invasion

When fungi invade plants, cell-wall reinforcements (CWAs), composed of callose, lignin, cell-wall proteins, and reactive oxygen species, form within the cell wall to defend against fungal invasion and penetration [117,120,121,122]. The plant cell wall is an extracellular matrix that envelopes cells, gives them structure and shape, constitutes the interface with symbionts, and defends plants against external biotic and abiotic stress factors [123]. Callose and cellulose are fundamental components of the cell wall and are probably synthesized by distinct enzymes, callose synthase and cellulose synthase, respectively. Previous studies examined the distribution of callose synthase and cellulose synthase in tobacco (*Nicotiana tabacum*) pollen tubes in relation to the dynamics of actin filaments, microtubules [118,123]. In tobacco pollen tubes, actin filaments and endomembrane dynamics are critical for the distribution of callose synthase and cellulose synthase, indicating that these enzymes are transported via Golgi bodies and/or vesicles moving along actin filaments; conversely, microtubules play a key role in positioning callose synthase in distal regions and around callose plugs [118].

Plants utilize polarized, cytoskeleton-guided exocytosis to deliver defense materials to infection sites [124,125,126]. In various plant–pathogen interaction systems, actin filaments radially converge near infection sites and assemble into highly stable dome-shaped patches beneath fungal penetration points [127,128]. Notably, during pathogen infection, the aggregation and increased density of microfilament bundles within plant cells are considered important signals for plants to perceive pathogen invasion [129,130] (Figure 2B). It has been reported that numerous factors regulate the formation of these microfilamentous plaques (Figure 2B). In the following section, we will discuss these factors in detail.

The actin cytoskeleton plays a critical role in plant immunity, particularly during pathogen invasion. Previous studies have demonstrated that two major actin-nucleating systems, the ARP2/3 complex and formins, are important for *Arabidopsis* penetration resistance to fungal invasion [72,73,74,119]. Specifically, cell-wall deposition is severely impaired in *arp2/3* mutants or in *Arabidopsis* FORMIN1 (AtFH1) knockdown lines, leading to enhanced susceptibility to powdery mildew [73]. These findings suggest the importance of coordinated actin nucleation in cell wall-associated defense. Furthermore, actin-related protein complex 4 (ARPC4), a member of the Arp2/3 complex of actin-binding proteins, also contributes critically to *Arabidopsis* penetration resistance against fungal invasion [72]. In *Arabidopsis*, mutation of ARPC4 disrupts microfilament organization and callose deposition, which in turn increases susceptibility to the necrotrophic fungus *Sclerotinia sclerotiorum* [72]. And, in tomato, the *ARPC3* gene, encoding a subunit protein of the Arp2/3 complex, is significantly upregulated when an incompatible host is involved in pathogen interaction, suggestive of a role for ShARPC3 in plant defense signaling and immunity [119]. Together, these observations indicate that the ARP2/3 complex appears to function broadly in both cell-wall reinforcement and callose deposition during immune responses. On the other hand, Sun et al. [74] demonstrate that AtPRF3 is an unconventional profilin isoform with an N-terminal extension, which causes protein oligomerization and inhibits formin-mediated actin assembly in *Arabidopsis*. And the *prf3 Arabidopsis* plants show higher sensitivity to the bacterial flagellum peptide in both the plant growth and ROS responses [74]. Moreover, AtPRF3 regulates PAMP-triggered immune responses, which in turn modulate AtPRF3 degradation [74].

The actin-depolymerizing factor (ADF) family plays a pivotal role in plant immune responses by dynamically remodeling the microfilament cytoskeleton (Figure 2B). In wheat, *TaADF7* expression is upregulated upon inoculation with the stripe rust pathogen (Pst: *Puccinia striiformis* f. sp. *tritici*) [79]. Silencing of *TaADF7* impaired defense responses, as evidenced by reduced expression of pathogenesis-related 1 (*PR1*), decreased accumulation of reactive oxygen species (ROS), and weakened hypersensitive response (HR). Notably, HR was alleviated, and the expression of *PR1*, a marker of the salicylic acid (SA) pathway, was significantly reduced upon *TaADF7* silencing. Furthermore, *TaADF7* was strongly induced by exogenous SA treatment, suggesting a mutual relationship between *TaADF7* and SA signaling within microfilament cytoskeleton dynamics during immune responses [79]. Suppression of *AtADF1* and *AtADF4* enhances resistance to the adapted powdery mildew fungus *Golovinomyces orontii* [82]. Inada et al. [82] report that the enhanced resistance of *adf4* and ADF1-4 knockdown plants (ADF1-4Ri) was associated with the accumulation of hydrogen peroxide and cell death specific to G. orontii-infected cells [82]. Moreover, the calcium-dependent protein kinase CPK3 enhances resistance to bacterial infections by phosphorylating ADF4, inactivating its depolymerizing function, and stabilizing actin filaments [83]. In cotton, downregulation of GhADF6 after *Verticillium dahliae* infection stabilizes actin filaments and improves fungal tolerance [93].

By contrast, loss of AtADF4 confers on *Arabidopsis* enhanced susceptibility to *P. syringae* expressing AvrPphB [80]. Tian et al. [80] reported that AtADF4 was identified as specifically required for AvrPphB-triggered immunity. AtADF4 binds G-actin and inhibits nucleotide exchange, acting as a bona fide actin-depolymerizing factor. Critically, AtADF4 transmits defense signals by modifying the actin cytoskeleton, rather than by blocking pathogen entry [80]. In contrast, Henty-Ridilla et al. [81] provide genetic and cytological evidence that inhibition of ADF4 during plant innate immune signaling regulates actin dynamics in order to execute key events associated with PTI, such as cell-wall fortification and transcriptional activation of defense gene markers [81].

Beyond classical ADFs, the microtubule-destabilizing protein MDP25 also participates in actin-mediated immunity. Upon perception of flg22, MDP25 translocates from the plasma membrane to the cytoplasm, where it interacts with the outer mitochondrial membrane (OMM) protein voltage-dependent anion channel 3 (VDAC3) at mitochondria-associated actin contact sites. The MDP25/VDAC3 complex coordinates actin reorganization to promote mitochondrial fusion and elongation, facilitating metabolic exchange and enhancing the production of ATP and mitochondrial ROS, which are the key components of an effective immune response [94]. Actin filament bundles are higher-order cytoskeletal structures that are crucial for the maintenance of the immune response. Zou et al. [96] found MPK3/6 (mitogen-activated protein kinases) phosphorylates VLN3 to remodel actin and activate stomatal immunity, which is critical for bacterial resistance in *Arabidopsis* [96]. In summary, the regulation of the actin cytoskeleton is a focal point in the molecular interaction between plants and pathogens.

### 3.2. Cytoskeleton Remodeling Under Abiotic Stresses

#### 3.2.1. Cytoskeletal Responses to Temperature Stresses

High temperature affects the growth and development of plants and results in physiological changes and intracellular signaling responses in plants [131,132]. Accumulating evidence indicates that heat stress also severely disturbs the organization of the cytoskeleton in plant cells. For instance, Müller et al. [6] reported that cytoskeletal components in *Arabidopsis* exhibit highly dynamic behaviors under high temperature, undergoing transient depolymerization and disassembly before fully recovering within 1–3 h at 20 °C [6]. Moreover, in tobacco pollen tubes, heat stress impairs proteins that bind and involve in cell-wall synthesis, such as sucrose synthase, which further disrupts cytoskeletal architecture and consequently impairs vesicular transport and cell-wall deposition [5]. Consistent with these observations, Malerba et al. [133] reported that high temperature also induces actin filament depolymerization in tobacco BY2 suspension cells, leading to the formation of fragmented microfilaments [133].

**Figure 3:**
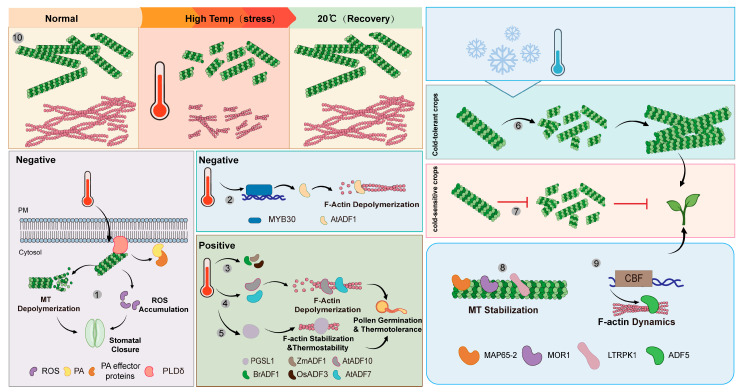
Cytoskeleton-mediated adaptation to temperature stress. ① Heat stress activates plasma membrane-localized PLDδ, which catalyzes the production of phosphatidic acid (PA) and induces intracellular ROS accumulation, thereby initiating early signaling responses to heat stress [138,139,141]. ② Heat induces MYB30, which promotes ADF1 transcription, but overall heat reduces ADF1 expression. ADF1 depolymerizes actin filaments and negatively regulates thermotolerance in *Arabidopsis* [86]. ③ ZmADF1 is upregulated under high temperature and participates in actin cytoskeleton rearrangement [142]. BrADF1 functions similarly to AtADF1 and promotes F-actin depolymerization [86]. OsADF3 [143] is heat-induced in heat-tolerant rice and is involved in the response to high-temperature stress [142]. ④ The highly thermostable proteins AtADF7 and AtADF10 promote the dynamic turnover of F-actin in pollen grains under high temperature, thereby enhancing pollen germination capacity and heat tolerance under heat stress [144]. ⑤ Heat-stable actin-binding protein PGSL1 binds and stabilizes actin filaments, which play an essential role in pollen thermotolerance [104]. ⑥ Under cold stress, microtubules in cold-tolerant crops undergo dynamic rearrangement, forming stable cortical microtubule arrays and thereby mediating cold adaptation [145]. ⑦ Under cold stress, microtubules in cold-sensitive crops depolymerize, disrupting cell structure and leading to cold stress damage [146]. ⑧ AtMAP65-2 [57], LTRPK1 [147], and MOR1 [148] participate in regulating plant cold tolerance by stabilizing cortical microtubules, modulating microtubule stability and dynamics. ⑨ The actin-binding protein ADF5 is activated by CBF transcription factors and enhances freezing tolerance by stabilizing actin dynamics and endocytosis [90]. ⑩ Under high temperature, *Arabidopsis* cytoskeletal components transiently depolymerize and disassemble, then fully recover within 1–3 h at 20 °C.

When plants are subjected to temperature stresses, phospholipase D (PLD) is activated to generate phosphatidic acid (PA) and a head group [134,135]. PA is emerging as an important signaling lipid in all organisms by binding effector proteins and recruiting them to a membrane, which regulates the proteins’ activities in cellular pathways [136,137]. Heat shock treatment of wild-type *Arabidopsis* cotyledons stimulated ROS production, which disrupted microtubule organization and induced stomatal closure, whereas this process was significantly compromised in *pldδ* mutants [138]. Heat shock-induced PA elevation via PLD activation, together with PIP2, regulates the actin cytoskeleton: PA is involved in cytoskeletal modulation [139]. Heat shock also activates PLD to elevate cellular PA levels, which may result in opening of downstream ion channels [139,140]. Zhang et al. [141] showed that phospholipase Dδ (PLDδ) was associated with the PM and co-localized with microtubules and PLDδ also bound to microtubules in vitro, resulting in microtubule disorganization [141]. When the *Arabidopsis* seedlings were treated with heat shock, PLDδ transiently was activated but without any change in its PM localization, triggering microtubule dissociation from PM, depolymerization and seedling death [141]. These effects are significantly alleviated in *pldδ* knockout mutants. Thus, the PM-associated PLDδ negatively regulates plant thermotolerance via destabilizing cortical microtubules (Figure 3) [141].

Profilins and ADFs respond strongly to heat stress, suggesting that depolymerization of the MFs induced plant sensitivity to heat stress and polymerization of the MFs improved the ability of plants to withstand heat stress [149]. AtADF1 is an important regulator of actin filaments during high-temperature adaptation in *Arabidopsis*, and its expression is directly regulated by MYB30. Chinese cabbage BrADF1, which shares high homology with AtADF1, also participates in thermal adaptation through a similar mechanism to AtADF1 [86]. In addition, *ZmADF1* was also significantly upregulated in heat stress [142]. Similarly, the transcription level of OsADF3 in heat-tolerant rice varieties is higher [143]. Additionally, the heat-stable actin-binding protein PGSL1 (pollen germination sensitive to LatB protein 1) binds and stabilizes actin filaments, which plays an essential role in pollen thermotolerance [104]. The evolution of high thermal stability in ADFs, such as AtADF7 and AtADF10, might play a pivotal role in the origin and evolution of *Arabidopsis*’s adaptation to high temperatures, as these ADFs exhibit high thermal stability and promote the dynamic turnover of actin filaments in pollen grains at high temperatures, thereby enhancing pollen germination under such conditions [144].

Turning to the microtubule network at the cytoskeletal level, it not only plays a crucial role in temperature tolerance, but also responds sensitively to temperature changes [145,150]. Microtubules in the cortex of the root elongation zone disassembled rapidly in response to a cold shock of –7 °C and reassembled upon rewarming to 25 °C in Chinese winter wheat (*Triticum aestivum* L.) [150]. The microtubules acquired resistance against this cold shock in response to cold acclimation in chilling, but non-freezing, temperature or after a treatment with abscisic acid (ABA). A rapid, but transient partial disassembly in the tolerant cultivars preceded the formation of cold-stable microtubules and the recovery of growth rate in winter wheat (*Triticum aestivum* L.) [145]. However, this transient disassembly was absent in the sensitive cultivar [145]. These results indicate that the transient disassembly and rapid recovery of microtubules are crucial for breeding cold-tolerant crops. Previous studies have suggested that the structural arrangement and also the dynamics of MTs are believed to be controlled by MAPs such as MAP65, MOR (*MICROTUBULE ORGANIZATION 1*), and katanin [151,152,153]. AtMAP65-2 greatly stabilized MTs that were subjected to low-temperature treatment in vitro, suggesting AtMAP65-2 promotes cold tolerance through strong stabilization of cortical MTs [57]. The rice low-temperature stress response protein kinase 1 (LTRPK1) was shown to regulate the stability and dynamics of microtubules under low-temperature stress [147]. Furthermore, MICROTUBULE ORGANIZATION 1 (MOR1) is mobilized to stabilize microtubule ends and participate in reorganization under extreme cold [148].

Low temperature restricts plant growth, and the actin cytoskeleton plays a central role in cold stress responses, though limited research has been conducted (Figure 3). Zhang et al. [90] show that in *Arabidopsis*, the actin-binding protein *Arabidopsis* actin depolymerizing factor 5 (ADF5) is transcriptionally activated by C-repeat binding factor (CBF) transcription factors via direct binding to the *ADF5* promoter. Cold-induced *ADF5* expression stabilizes actin dynamics and endocytosis, thereby enhancing freezing tolerance [90]. In support, Xu et al. [91] report that overexpression of wheat *TaADF16*, another actin depolymerizing factor, increases freezing tolerance in transgenic *Arabidopsis*, likely through improved ROS scavenging and osmotic regulation [91]. In addition, heat shock proteins (HSPs) are molecular chaperones that play a crucial role in plant temperature tolerance [154,155]. For example, NtHSP90 directly binds to microtubules and the inhibition of Hsp90 by geldanamycin (GDA) severely impairs MT re-assembly after cold-induced depolymerization [60]. Together, these findings highlight the conserved and critical role of actin cytoskeleton remodeling in plant cold acclimation.

Species- or tissue-specific sensitivity to temperature stress differs. In *Arabidopsis* roots, actin filament disassembly was observed within 7–25 min at 42 °C [6], whereas in tobacco BY-2 cells a similar effect on actin filaments required a higher temperature of 50 °C (5 min) [133], suggesting that tobacco suspension cells may possess greater thermotolerance. Furthermore, the relative response rates of microtubules and actin filaments are not consistent between low and high temperatures. At 0 °C, radial actin filaments in the transvacuolar strand disappeared after 5 min, while microtubule disassembly was detected only after 20 min [156]. In *Arabidopsis* roots at 42 °C, actin filament disassembly (7–25 min) also occurred slightly earlier than microtubule disassembly (approximately 30 min) [6]. However, in tobacco BY-2 cells at elevated temperature, microtubule disassembly was observed at 42 °C after 30 min, whereas actin filament disassembly required 50 °C (5 min) [133]. Heat shock proteins (HSPs) are molecular chaperones that play a crucial role in plant temperature tolerance [154,155]. NtHSP90 directly binds to microtubules, and the inhibition of Hsp90 by geldanamycin (GDA) severely impairs MT re-assembly after cold-induced depolymerization [60].

#### 3.2.2. Cytoskeletal Dynamics Under Drought Stress

Drought stress triggers a series of profound physiological and morphological adaptations in plants [157], with stomatal closure [158] and root system architectural remodeling [88,159] being the most visible adaptive responses. Stomata close rapidly to reduce transportational water loss, while roots explore deeper soil moisture through elongation, directional growth, or increased root hair density [159,160]. Stomatal closure under drought stress is also driven by the coordinated reorganization of the microtubule and actin cytoskeletons within guard cells [3,66,161,162].

**Figure 4:**
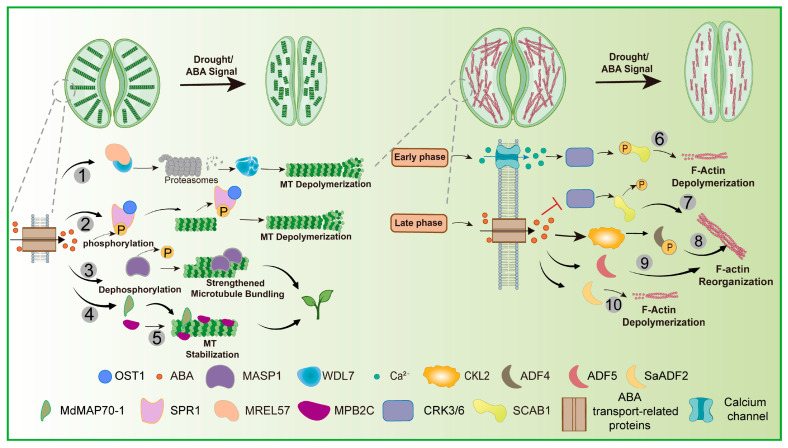
Coordinated cytoskeletal remodeling mediates stomatal to drought stress. ① MREL57-WDL7 module in microtubule disassembly and stomatal closure in response to drought stress and ABA [66]. ② Drought-activated ABA signaling in guard cells causes OST1 to phosphorylate SPR1, promoting its dissociation from microtubules, thereby inducing microtubule depolymerization and stomatal closure [65]. ③ ABA signaling enhances MASP1-mediated microtubule stability [161]. ④ Overexpression of the apple microtubule-associated protein gene MdMAP70 1 in tomato increased drought resistance [63]. ⑤ Overexpressing transgenic plants of MPB2C enhanced drought tolerance [162]. ⑥ ABA/osmotic stress-induced Ca^2+^ influx activates CPK3/6 to phosphorylate SCAB1, reducing its actin affinity and triggering rapid depolymerization to initiate stomatal closure [3]. ⑦ Sustained ABA signaling inhibits CPK3/6, leading to SCAB1 dephosphorylation, which restores its F-actin-binding ability and promotes F-actin rebundling to provide mechanical stability for closed stomata [3]. ⑧ ABA signals activate the kinase CKL2, which phosphorylates ADF4, suppressing the actin-depolymerizing function of ADF4, thereby stabilizing actin filaments and further promoting stomatal closure [85]. ⑨ Drought/ABA upregulate ADF5 through DPBF3, and ADF5 promotes stomatal closure via actin remodeling, enhancing drought tolerance [89]. ⑩ Rice overexpressing SaADF2 maintains better actin filament organization and chloroplast grana structure, along with higher photosynthesis and yield, under drought stress [92].

Microtubule depolymerization is an early cellular event facilitating stomatal closure (Figure 4). Recently, Dou et al. [66] revealed that the ubiquitin-26S proteasome system (UPS) promotes microtubule disassembly by degrading the microtubule-stabilizing protein WDL7 via the MREL57 (Microtubule-Related E3 Ligase target 57) E3 ligase. This process is essential for ABA-induced stomatal closure and plant adaptation to drought stress. In *mrel57* mutants, ABA-induced microtubule disassembly and stomatal closure are impaired, but these defects can be rescued by reducing WDL7 expression [66]. Beyond the degradation of microtubule stabilizers, plants also actively reinforce their cytoskeleton under stress. A key mechanism involves the microtubule-associated protein 1 (MASP1). The drought hormone ABA promotes the dephosphorylation of MASP1, a modification that prevents its proteasomal degradation. Consequently, MASP1 protein abundance increases significantly. This accumulation enhances MASP1′s microtubule-bundling activity, leading to a stabilized cytoskeleton that supports cellular integrity and confers drought tolerance [161]. Similarly to MASP1, another microtubule-associated protein, MPB2C, has also been implicated in drought tolerance. Endogenous MPB2C localized in punctae at cortical microtubules, suggesting its interaction with distinct sites at microtubules [163]. GFP-AtMPB2C-overexpressing transgenic plants were characterized by clockwise twisted leaves, clustered stomata, and enhanced drought tolerance, suggesting that AtMPB2C is involved in the alignment of cortical microtubules, the patterning of stomata [162]. In summary, drought elicits a dual regulation of the microtubule cytoskeleton, coordinating both microtubule destabilization and stabilization pathways to execute stomatal movement.

Drought stress triggers ABA signaling in guard cells and induces stomatal closure, a process accompanied by cortical microtubule disassembly [164]. Wang et al. [65] identified the microtubule-associated protein SPIRAL1 (SPR1) as a substrate of the ABA signaling core component OPEN STOMATA 1 (OST1). OST1 interacts with and phosphorylates SPR1 at Ser6, promoting its dissociation from microtubules and facilitating microtubule disassembly. Compared with wild-type plants, the spr1 mutant showed greater water loss and reduced ABA responses, including impaired stomatal closure and microtubule disassembly in guard cells; these phenotypes were restored by introducing a phospho-active form of SPR1, demonstrating that SPR1 positively regulates ABA-induced microtubule disassembly in an OST1-dependent manner [65]. In another study, Li et al. [63] showed that overexpression of the apple microtubule-associated protein gene *MdMAP70-1* in tomato increased relative water content, proline and soluble protein levels, and the activities of superoxide dismutase, peroxidase and catalase under drought stress, while reducing relative electrolyte conductivity and malondialdehyde content, thereby enhancing drought resistance [63].

Meanwhile, the actin cytoskeleton undergoes rapid reorganization upon drought (Figure 4). A drought-triggered rise in cytosolic calcium activates the kinases CPK3/6 in guard cells, which promptly phosphorylate the actin-binding protein SCAB1 (Stomatal Closure-Related Actin-Binding Protein 1), reducing its affinity for F-actin and disrupting its stabilizing function, leading to actin depolymerization and providing the mechanical flexibility required for stomatal closure [3]. In parallel, sustained ABA inhibits CPK3/6, leading to SCAB1 dephosphorylation and F-actin rebundling, which provides mechanical stability for closed stomata [3]. In a parallel pathway, drought and ABA signals activate the kinase CKL2, which phosphorylates the actin-depolymerizing factor ADF4, suppressing the actin-depolymerizing function of ADF4, thereby stabilizing actin filaments and further promoting stomatal closure [85]. Sengupta et al. [92] also found rice transgenics constitutively overexpressing *SaADF2* (*SaADF2*-OE) showed better growth, relative water content, and photosynthetic and agronomic yield under drought conditions than wild-type (WT) [92]. In addition, *Arabidopsis* ADF5 also plays an important role in response to drought and ABA signaling. Qian et al. [89] demonstrated that drought stress and ABA treatment induce the expression of neofunctionalized *Arabidopsis ADF5*, which encodes an actin depolymerizing factor with F-actin bundling activity. Loss-of-function mutations in *ADF5* increased water loss from detached leaves, reduced post-drought survival, and delayed stomatal closure by impairing actin cytoskeleton remodeling. The ABF/AREB (ABA-responsive element binding factor) transcription factor DPBF3 (Dc3 Promoter-Binding Factors) was shown to bind the ABA-responsive element (ACGT/C) in the *ADF5* promoter and activate its transcription, placing ADF5 as a downstream target of the drought/ABA signaling pathway in the regulation of stomatal closure [89].

#### 3.2.3. Cytoskeletal Dynamics Under Salt Stress

Early studies have shown that salt stress can affect the organization of microtubules, and a wealth of research evidence has accumulated regarding the scientific question of whether microtubules play an active regulatory role in plant salt stress tolerance. For example, Wang et al. [165] showed that cortical microtubules depolymerized then reorganized themselves under salt stress, and both processes are important for a plant’s ability to withstand salt stress [165]. Notably, stabilizing microtubules with paclitaxel increased seedling death, whereas disrupting them with oryzalin or propyzamide improved survival, suggesting that controlled depolymerization and subsequent reorganization are critical for salt tolerance [165]. This counterintuitive observation suggested that active microtubule turnover, rather than mere stability, is essential for salt tolerance.

On one hand, some regulators promote microtubule stability to confer tolerance. For instance, salt stress signaling activates phospholipase Dα1 (PLDα1) to produce phosphatidic acid (PA), which binds to MAP65-1; this interaction enhances microtubule stability and promotes salt tolerance [53]. The armadillo repeat-containing protein CSI1 (cellulose synthase-interactive protein 1) binds microtubules and stabilizes them; under dehydration (a component of salt stress), CSI1 dynamically changes to facilitate microtubule depolymerization and reorganization, which is crucial for anther development [67]. Moreover, sustained cellulose synthesis conferred by (cellulose synthase 6) CESA6 and CSI1 is important for salt stress tolerance [68].

On the other hand, controlled microtubule depolymerization is equally important. Microtubule-destabilizing protein 25 (MDP25, also known as PCaP1) mediates microtubule depolymerization under salt stress: Elevated cytosolic Ca^2+^ causes MDP25 to partially dissociate from the plasma membrane, thereby promoting microtubule depolymerization. Knockout of *MDP25* improved microtubule reassembly and integrity under prolonged salt stress and exhibited a higher seedling survival, revealing a role for MDP25 in regulating microtubule organization under salt treatment by affecting microtubule dynamics [51]. In hybrid poplar, salinity stress induces the expression of PagPCaP1a and triggers its calcium-dependent phase separation to form PagPCaP1a condensates; this process rapidly depolymerizes microtubules, representing an additional regulatory layer for stress acclimation [71]. Another microtubule-associated protein, ATKATANIN1 (AtKTN1), plays a complex role in salt stress response: Overexpression of AtKTN1 reduces salt tolerance, whereas knockout of *AtKTN1* enhances tolerance at early stages but decreases at later stages [70], suggesting that plant salt tolerance relies on a delicate balance between proper microtubule organization and dynamics. Similarly, in rice, the microtubule-encoding gene *OsTUB1* interacts with Kinesin13A to stabilize microtubule organization and sustain plasma membrane-localized Na^+^ transporter OsHKT1;5, thereby protecting plants from salt stress [52]. Additionally, the RING E3 ligase microtubule-targeting domain 1 (OsRMT1) targets microtubules via its N-terminal domain; its degradation is inhibited under salt stress, and its overexpression increases salt tolerance, likely by modulating levels of target proteins [62]. By contrast, another rice RING E3 ligase, OsMAR1 (Oryza sativa microtubule-associated RING finger protein 1), negatively regulates salt stress responses by interacting with and degrading the cytosolic protein OCPI2 (O. sativa chymotrypsin protease inhibitor 2) [61].

**Figure 5:**
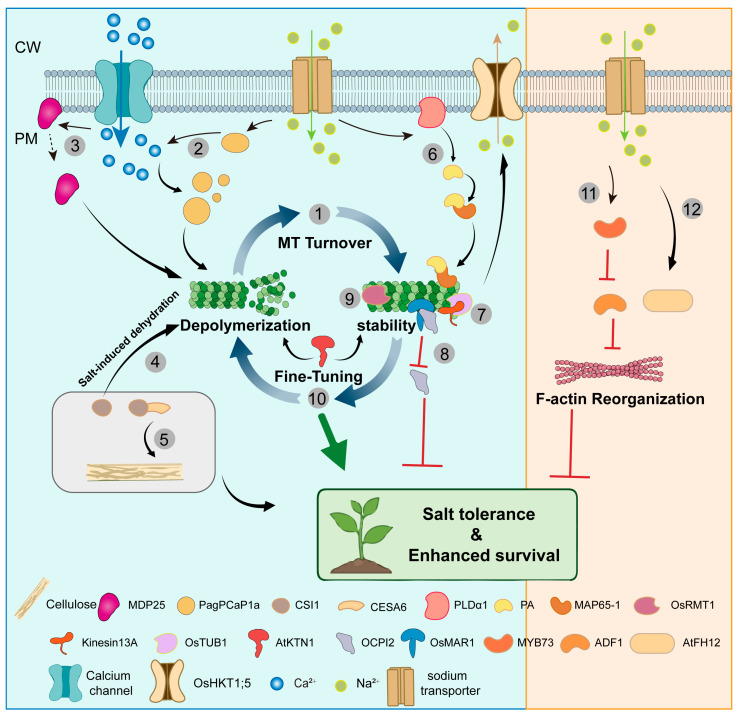
Coordinated cytoskeletal remodeling to salt stress. ① Cortical microtubules depolymerized then reorganized themselves under salt stress, and both processes are important for a plant’s ability to withstand salt stress [165]. ② In hybrid poplar, salt stress induces PagPCaP1a, which forms Ca^2+^-dependent condensates via phase separation, rapidly depolymerizing microtubules and providing an additional stress adaptation pathway [71]. ③ Elevated cytosolic Ca^2+^ causes partial dissociation of MDP25 (PCaP1) from the plasma membrane; free MDP25 then enters the cytosol and mediates microtubule depolymerization. [51]. ④ CSI1 binds and stabilizes microtubules, but under salt-induced dehydration stress, it dynamically shifts to promote microtubule depolymerization and reorganization [67]. ⑤ CSI1 interacts with cellulose synthase CESA6 to maintain continuous cellulose synthesis, enhancing salt tolerance in plants [85]. ⑥ Salt stress signaling activates phospholipase Dα1 (PLDα1) to produce phosphatidic acid (PA), which binds to MAP65-1; this interaction enhances microtubule stability and promotes salt tolerance [53]. ⑦ OsTUB1 interacts with kinesin13A to stabilize microtubules and maintain plasma membrane localization of the Na^+^ transporter OsHKT1;5, promoting Na^+^ efflux for salt tolerance [52]. ⑧ The microtubule-associated RING-type E3 ligase OsMAR1 negatively regulates salt stress response in rice by binding and degrading the cytosolic protein OCPI2 [61]. ⑨ Salt stress inhibits degradation of the microtubule-targeted RING-type E3 ligase OsRMT1, leading to its accumulation, which enhances rice salt tolerance by regulating target protein levels [62]. ⑩ Overexpression of AtKTN1 reduces salt tolerance, whereas knockout of AtKTN1 enhances tolerance at early stages but decreases it at later stages [70]. ⑪ MYB73 binds the ACCTAC motif in the ADF1 promoter and represses its expression. Thus, ADF1 regulates actin organization to promote salt tolerance as a downstream target of the negative regulator MYB73 [54]. ⑫ AtFH12 (formin homology 2) is induced by NaCl but has negligible phenotypic effects under salt stress, suggesting AtFH12 is probably involved in salt stress [78].

In contrast to the extensive body of work on microtubules, the role of actin filaments under salt stress remains largely unexplored, with limited studies having briefly addressed this aspect. Among these, salt stress has been shown to induce the expression of ADF1. The *adf1* mutants show reduced survival, increased actin cables, and decreased filament density; overexpression has opposite effects. MYB73 binds the ACCTAC motif in the *ADF1* promoter and represses its expression [54]. AtFH12 (formin homology 2) is induced by NaCl but has negligible phenotypic effects under salt stress, suggesting AtFH12 is probably involved in salt stress [78]. Together, these results underscore the central importance of the cytoskeleton in mediating plant adaptation to salt stress (Figure 5).

#### 3.2.4. Light Regulation of Cytoskeletal Dynamics

The cytoskeleton serves as a central hub through which plants and macroalgae translate light signals into directional growth and morphological adaptations [166,167]. Previous studies have demonstrated that cortical microtubules (CMTs) affect the axis of cell elongation and are thus indispensable for normal morphogenesis [151]. Transversely aligned CMTs facilitate cell elongation, promoting long hypocotyl growth, whereas a longitudinal orientation inhibits lateral expansion, resulting in shorter hypocotyls [168,169]. During light-regulated hypocotyl growth, CMT dynamics are precisely tuned. Under high-intensity light, [Ca^2+^] cyt levels significantly and transiently increase, which triggers the microtubule-destabilizing protein MDP25 to dissociate from the plasma membrane and promote microtubule depolymerization, thereby inhibiting hypocotyl elongation [170,171]. Furthermore, the abundance of microtubule-associated proteins is regulated by the ubiquitin-26S proteasome pathway mediated by the E3 ubiquitin ligase COP1. In darkness, the E3 ubiquitin ligase COP1 enters the nucleus and induces the polyubiquitination and degradation of WDL3, leading to the depolymerization of cortical microtubules, thereby facilitating cell elongation. In contrast, light inactivates COP1 via activated photoreceptors, leading to the stabilization and accumulation of WDL3. Accumulated WDL3 binds to and hyper-stabilizes microtubules, reducing their turnover and flexibility. This rigidified microtubule array restricts the reorientation of cellulose synthase complexes and thus suppresses directional cell expansion, ultimately inhibiting cell elongation growth [7,8]. In addition, studies have also reported that katanin can be activated by blue light, which cuts microtubules at crossover points to reorganize them into longitudinal arrays. This reorganization is essential for phototropic responses [166].

In addition, a growing body of research has revealed that the actin cytoskeleton is dynamically remodeled by light, affecting both organelle motility and cell expansion. Wen et al. [56] found that strong blue light activates Phototropin 2 (Phot2), which recruits and activates Protein Phosphatase 2A (PP2A). PP2A then dephosphorylates specific actin-binding proteins (ABPs), leading to the polarized assembly of dynamic actin filaments alongside chloroplasts [56]. Myosin motors subsequently transport chloroplasts along these newly formed actin tracks, ultimately driving chloroplast movement away from the high-light area (i.e., the avoidance response) [56]. However, when *Arabidopsis* is grown in darkness, loss of 14-3-3 λ results in hyperphosphorylation and constitutive inactivation of ADF1, leading to excessively stabilized actin filaments and unrestrained hypocotyl elongation [172]. Besides the effects of blue light on actin filaments, ultraviolet light (UV) also exerts regulatory effects on them. UV-B inhibited *Arabidopsis* hypocotyl elongation by reorganizing actin filaments from bundles to a loose arrangement [55].

**Figure 6:**
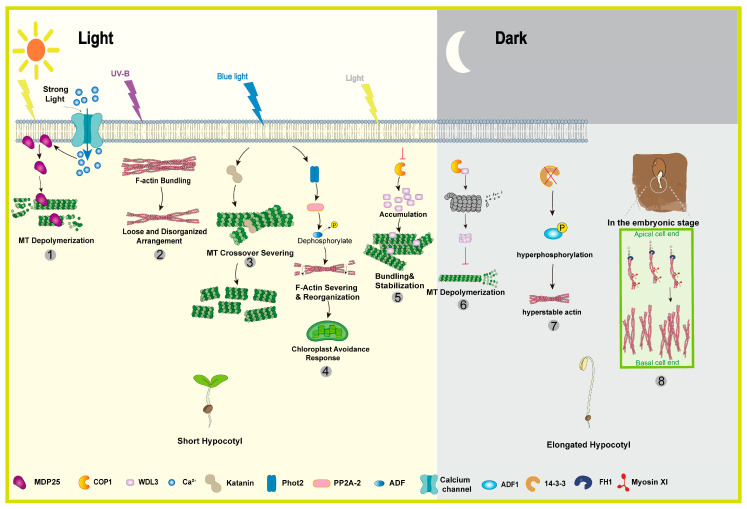
Cytoskeletal regulators’ response to light. ① Strong light-induced Ca^2+^ influx triggers MDP25 translocation from the cell membrane to the cytoplasm, where it depolymerizes microtubules to inhibit hypocotyl elongation [170]. ② UV-B radiation induces disordered actin bundling, which disrupts cell-wall deposition and inhibits hypocotyl growth [55]. ③ Blue light activates katanin to sever microtubules at crossovers, driving a transverse-to-longitudinal reorientation that limits lateral expansion [166]. ④ Blue light-activated Phot2 stimulates PP2A-2 phosphatase to regulate actin remodeling, ultimately driving chloroplast light avoidance movements [56]. ⑤ Light suppresses COP1 activity, thereby permitting accumulation of the microtubule stabilizer WDL3, which restricts hypocotyl growth by promoting microtubule bundling [8]. ⑥ In darkness, COP1 degrades WDL3, allowing WDL3-mediated microtubule stabilization to prevent excessive hypocotyl elongation [7]. ⑦ Loss of 14-3-3 λ leads to hyperphosphorylation of ADF1, which disrupts actin dynamics and enhances hypocotyl growth in the dark [172]. ⑧ A formin- and myosin XI-dependent actin array underpins polarized elongation in the embryonic hypocotyl [173].

During *Arabidopsis* seed germination, a polarized actin array forms at the basal end of hypocotyl cells to drive axial elongation. This array is assembled through two coordinated steps: formin 1 nucleates new actin filaments at the basal membrane, while apically localized myosin XI motors exert directional force to transport and align these filaments toward the basal region. This process establishes parallel actin cables that channel growth materials and provide the structural framework for rapid cell elongation [173]. In summary, plants integrate light cues with cytoskeletal dynamics through a sophisticated, multi-layered regulatory network (Figure 6). This network converges on the precise spatiotemporal control of both microtubule and actin organization, ultimately translating environmental light signals into appropriate developmental morphogenesis.

## 4. Concluding Remarks and Future Perspectives

### 4.1. Cytoskeleton Dynamics in Stress Signaling

The cytoskeleton serves not only as a structural scaffold but also as a dynamic signaling integrator during stress adaptation. Microtubule-associated proteins (MAPs) and actin-binding proteins (ABPs) can function as molecular hubs that converge multiple signaling pathways, including light, temperature, salt stress, and hormonal signals, onto cytoskeletal remodeling [10,11,174]. Under environmental stress, the dynamic remodeling of the cytoskeleton is far more important than the simple presence of its components. Under heat stress, actin filaments (AFs) respond faster but recover more slowly than microtubules (MTs): AFs depolymerize or bundle at 35–42 °C, whereas MTs only disassemble above 40 °C, showing slower initial changes but faster recovery [6]. This kinetic difference suggests a division of labor: rapid AF remodeling serves as an early signaling platform, while MTs maintain structural integrity.

Importantly, under pathogen stress, actin turnover (not just presence) affects immune signaling [80]. TaADF7 maintains actin dynamics to promote the SA pathway and HR; its knockdown stabilizes actin and blocks SA signaling even though actin remains present [79]. In contrast, in *Arabidopsis* ADF1-4 silenced lines, the formation of ordered actin bundles, which may represent a distinct dynamic state, still permits SA-dependent ROS and HR [82], suggesting that not all forms of actin stabilization impair SA signaling. By contrast, in ADF4 mutants, actin bundling does not activate SA but instead ectopically activates the JA pathway [80]. This differential outcome indicates that SA signaling requires continuous actin turnover, whereas JA signaling favors actin stabilization or bundling. In addition to its signaling roles, actin can also act as a physical barrier. For instance, ARP2/3 and formins form dense networks that block pathogen entry [73]. Consistent with the notion of context-dependent actin dynamics, ADF4 also exhibits opposing requirements in different immune settings. While Tian et al. [80] showed that ADF4 is required for AvrPphB-triggered gene-for-gene resistance [80], Henty-Ridilla et al. [175] found that inhibition of ADF4 promotes PTI-associated events [175]. These contrasting observations suggest that ADF4 functions differently depending on the immune context. Plants switch defenses according to pathogen lifestyle, and pathogens such as *Pseudomonas syringae* exploit this by producing coronatine to mimic JA and suppress SA [80]. Taken together, cytoskeletal adaptation does not rely on a single component but rather on the coordinated dynamics of actin filaments (and potentially microtubules), as well as on cross-talk between distinct immune pathways, to underpin precise stress responses. Future work should investigate how plants coordinately regulate actin and microtubule dynamics under combined stresses, and whether the differential temperature thresholds observed in vitro are relevant under natural fluctuating environments.

### 4.2. Actin–Microtubule Interactions

Cross-talk between microtubules (MTs) and actin filaments (AFs) is crucial for numerous cellular processes. MTs restrict plasma membrane protein diffusion and organize receptor polarity, whereas AFs drive clathrin-mediated endocytosis and vesicle transport [20,21].

Increasing evidence shows that some “shared proteins” interact with both cytoskeletal systems. For example, PCaP1/MDP25 was initially identified as an MT-destabilizing protein [170], but later found to sever actin filaments in a Ca^2+^-dependent manner, and it also functions in immunity by coordinating actin remodeling and mitochondrial dynamics [94]. PCaP2/MAP18, which binds microtubules and inhibits tubulin polymerization to regulate directional cell growth in roots and leaf pavement cells, while its Ca^2+^-dependent F-actin-severing activity guides pollen tube growth directionality [176,177,178]. Other bifunctional proteins further illustrate this functional duality. For instance, the *Arabidopsis* type II formin AtFH16 binds and bundles both actin filaments and microtubules in vitro, showing a preferential binding for microtubules over microfilaments; the FH1FH2 structure of AtFH16 does not efficiently nucleate actin polymerization but can bind and bundle both filament types [179]. Similarly, AtFH4 nucleates F-actin via its FH1FH2 domains and interacts with microtubules through a plant-specific GOE domain [180]. Beyond formins, the tobacco kinesin NtKCH (calponin homology domain) serves as a molecular cross-linker: during interphase, it associates with cortical microtubules, while a subfraction also co-localizes with perinuclear actin cables; in dividing cells, it accumulates at the pre-prophase band and phragmoplast [181]. Golgi movement is highly dependent on the actin cytoskeleton. In tobacco BY-2 cells, Golgi stacks exhibit stop-and-go movements that require intact AFs and myosin motors; MT depolymerization has only a mild effect [182].

In summary, MTs and AFs show a clear division of labor in plant cells, yet they also achieve molecular cross-talk via bifunctional shared proteins. How these proteins evolved their dual binding ability and how they coordinate seemingly independent signaling events (e.g., Ca^2+^ signaling and pathogen-induced Golgi transport) through the cytoskeletal network remain important questions.

### 4.3. Toward Designing Stress-Resilient Crops

Given the pivotal role of the cytoskeleton in stress responses, key MAPs and ABPs represent promising targets for molecular breeding and bioengineering. For example, modulating the expression or activity of specific regulators, whether through conventional breeding, transgenic approaches, or CRISPR-Cas9-mediated gene editing, can fine-tune the dynamic remodeling of microtubules and actin filaments under environmental stress. Such modifications could improve key architectural traits, including root system depth, stomatal regulation, and cellular integrity. In particular, CRISPR-Cas9 enables precise modification of genes encoding MAPs or ABPs, allowing for the engineering of an adaptive cytoskeletal rearrangement response directly in elite crop varieties. Translating this fundamental knowledge of cytoskeletal regulation into targeted crop improvement strategies offers strong potential for developing next-generation varieties with enhanced resilience to environmental challenges.

## 5. Conclusions

In summary, plants rely on the dynamic remodeling of the cytoskeleton to cope with both biotic and abiotic stresses, including temperature extremes, drought, salinity, and light fluctuations. This remodeling process is precisely orchestrated by MAPs and ABPs, which translate diverse stress signals into specific cytoskeletal rearrangements. Through these rearrangements, plants modulate downstream cellular events such as root architecture, stomatal movement, and cellular integrity, thereby achieving effective stress adaptation. The regulatory network involving post-translational modifications, cross-talk between microtubules and actin filaments, and spatiotemporal dynamics at the subcellular level has been partially characterized, underscoring the central role of the cytoskeleton as a hub for integrating stress signals. Overall, these insights provide a foundation for engineering crop varieties with improved cytoskeletal dynamics, thereby enhancing tolerance to combined stresses. 

## Figures and Tables

**Table 1:** Different MBP families with their specificity to distinct (a)biotic stress responses.

ABPs Types	Species	Proteins	Activities or Effects on Microfilament	(A)biotic Stress Responses
MAP65	*Arabidopsis thaliana*	AtMAP65-1	Microtubule organization	Promotes salt tolerance by stabilizing microtubules via PA–MAP65-1 interaction [53].
		AtMAP65-2	Stabilizes microtubules	Promotes cold tolerance through strong stabilization of cortical MTs [57].
		AtMAP65-3 /PLEIADE	Microtubule organization	Negatively interferes with plant defense against filamentous biotrophs [58].
	*Cucumis sativus* L.	CsaMAP65-1	Localized in microtubule and microfilament	*CsaMAP65-1* in leaves is significantly upregulated by cold stress, and this promotion is higher in cold-tolerant cultivar than intolerant cultivar [59].
		CsaMAP65-5	Localized in microtubule and microfilament	CsaMAP65-5 promotes salt tolerance [59].
Kinesin and kinesin-like protein	*Oryza sativa*	Kinesin 13-A	Promotes microtubule depolymerization	OsTUB1–Kinesin13A complex increases salt tolerance by stabilizing MT organization [52].
Chaperons or enzymes	*Nicotiana tabacum*	NtHsp90	Microtubule reorganization	The inhibition of *Hsp90* by geldanamycin (GDA) severely impairs MT re-assembly after cold-induced depolymerization [60].
RING finger protein	*Oryza sativa*	Microtubule-associated RING finger protein 1 (OsMAR1)		OsMAR1 has hypersensitivity phenotypes in *Arabidopsis* under high salt stress [61].
RING finger protein		RING finger protein with microtubule-targeting domain 1 (OsRMT1)		Overexpression of *OsRMT1* in *Arabidopsis* results in increased tolerance to salt stress [62].
Plant specific MAPs	*Apple Rootstock*	MdMAP70-1		Overexpression of *MdMAP70-1* gene in tomato can enhance the drought resistance of tomato [63].
Other proteins	*Arabidopsis thaliana*	SPIRAL1 (SPR1)	Plant-specific microtubule-localized protein	Accelerated SPR1 degradation is required for a fast MT disassembly response to salt stress and for salt stress tolerance [64]. And SPR1 positively regulates microtubule disassembly during ABA-induced stomatal closure [65].
Microtubule-destabilizing protein	*Arabidopsis thaliana*	MDP25	Microtubule organization	*mdp25* seedlings exhibited a higher survival rate under salt stress [51].
other	*Arabidopsis thaliana*	WAVE-DAMPENED2-LIKE7 (WDL7)	Stabilizes microtubules	The MREL57-WDL7 module regulates microtubule disassembly to mediate stomatal closure in response to drought stress and ABA treatment [66].
Other proteins	*Arabidopsis thaliana*	WAVE-DAMPENED2-LIKE5 (WDL5)	Stabilizes microtubules	Promotes ethylene-associated microtubule reassembly and plant salt stress tolerance [66].
Other proteins	*Arabidopsis thaliana*	Cellulose synthase-interactive protein1 (CSI1)	Stabilizes microtubules	*csi1-2* and *csi1-3* are all hyper-sensitive to salt stress [67,68].
Microtubule-destabilizing protein	*Arabidopsis thaliana*	MAP18/PCaP2	Destabilizes microtubules	*PPCaP2* plays an important and positive role in *Arabidopsis* water deficit tolerance by being involved in the response to both ABA and SA signals [69].
Other proteins	*Arabidopsis thaliana*	Microtubule-severing enzyme ATKATANIN1 (AtKTN1)	Severing microtubules	The OE-*AtKTN1* decreases tolerance to salt stress, whereas the knockout of *AtKTN1* increased salt tolerance in the early stage but decreased salt tolerance in the later stage [70].
Other proteins	*Arabidopsis thaliana*	Microtubule-Associated Stress Protein 1 (MASP1)	Microtubule organization	*OE-MASP1* enhances recovery of microtubule organization during drought acclimation [71].
Microtubule-destabilizing protein	*Populus*	PagPCaP1a (MDP25)	Destabilizes microtubules	*PagPCaP1a* condensates enhance the efficiency of microtubule depolymerization under salinity stress [71].

**Table 2:** Different ABP families with their specificity to distinct (a)biotic stress responses.

ABPs Types	Species	Proteins	Activities or Effects on Microfilament	(A)biotic Stress Responses
Actin nucleation factor	*Arabidopsis thaliana*	ARPC4	Actin nucleation	Positive to plant disease resistance by organizing cell-wall deposition [72].
	*Arabidopsis thaliana*	APRC2/3	Actin nucleation	The ARP2/3 complex and formins contribute to *Arabidopsis* penetration resistance to fungal invasion [73].
Actin nucleation factor	Tomato	ARPC3	Actin nucleation	Positive in plant defense signaling and immunity by inducing hypersensitive cell death and the generation of reactive oxygen [73].
Profilin	*Arabidopsis thaliana*	AtPRF3	Actin assembly	Profilin negatively regulates formin-mediated actin assembly to modulate PAMP-triggered plant immunity [74].
Profilin	*Arabidopsis thaliana*	AtPRF1	Actin nucleation	*Arabidopsis* profilin 1 mediates ATP-independent refolding of misfolded proteins under stress, such as biotic stressors such as salicylic acid (SA), jasmonic acid (JA), and bacterial pathogen exposure [75].
Profilin	*Arabidopsis thaliana*	AtPRF2		Oligomeric forms of AtPFN2 exhibit holdase-like molecular chaperone activity, which helps prevent protein aggregation under oxidative and heat stress [76,77].
Formin	*Arabidopsis thaliana*	AtFH12	Actin nucleation	*AtFH12* is induced by NaCl, producing only negligible phenotypic effects under salt stress [78].
Actin-Depolymerizing Factor (ADF)	wheat	TaADF7	Actin sever	Positive to defense responses [79].
Actin-Depolymerizing Factor (ADF)	*Arabidopsis thaliana*	ADF4	Actin sever	Positive to defense responses [80,81]; Negative to plant immunity by affecting the accumulation of hydrogen peroxide and cell death specific to G. orontii-infected cells [82,83]; Negative regulator of osmotic tolerance [84]; positive regulator of drought tolerance [85].
Actin-Depolymerizing Factor (ADF)	*Arabidopsis thaliana*	ADF1	Actin sever	Negative to plant immunity by affecting the accumulation of hydrogen peroxide and cell death specific to G. orontii-infected cells [82]; Negative regulator of heat tolerance [86]; positive regulator of salt tolerance [54].
Actin-Depolymerizing Factor (ADF)	*Arabidopsis thaliana*	ADF2	Actin sever	Positively regulates plant resistance to root-knot nematodes and negative regulator of osmotic tolerance [87].
Actin-Depolymerizing Factor (ADF)	*Arabidopsis thaliana*	ADF7	Actin sever	Positive regulator of osmotic tolerance [88].
Actin-Depolymerizing Factor (ADF)	*Arabidopsis thaliana*	ADF5	Actin filament bundling and stabilization	Positive regulator of drought tolerance [89].
Actin-Depolymerizing Factor (ADF)	*Arabidopsis thaliana*	ADF5	Actin filament bundling and stabilization	Promotes basic and acquired freezing resistance in *Arabidopsis thaliana* [90].
Actin-Depolymerizing Factor (ADF)	Wheat	TaADF16		*OE-TaADF16* increased the freezing tolerance of transgenic *Arabidopsis* [91].
Actin-Depolymerizing Factor (ADF)	Smooth cordgrass	SaADF2	Depolymerized F-actin filaments	*SaADF2* overexpression conferred drought tolerance in rice [92].
Actin-Depolymerizing Factor (ADF)	Cotton	GhADF6	Actin sever	Negative plant immunity: stabilizes actin filaments and improves fungal tolerance [93].
Microtubule-destabilizing protein	*Arabidopsis thaliana*	MDP25	Actin sever	Positive to plant immunity by actin reorganization to promote mitochondrial fusion [94].
Actin-bundling proteins	*Arabidopsis thaliana*	VILLIN1	Promotes actin bundle formation and stabilizes	The GL2-VLN1 pathway negatively responds to osmotic stress-induced root hair growth [95].
Actin-bundling proteins	*Arabidopsis thaliana*	VILLIN3	Dependent severing by Ca^2+^	Loss-of-function *vln3-1* and *vln3-2* mutants with bacterial pathogen *P. syringae* pv. tomato *DC3000* (DC3000) shows enhanced susceptibility to DC3000 compared with wild-type (WT) plants [96].
Actin-bundling proteins	*Gossypium hirsutum*	GhVLN4	Remodeling the actin cytoskeleton	*Arabidopsis* overexpressing GhVLN4 exhibited higher resistance to *V. dahlia* [97].
Actin-bundling proteins	*Arabidopsis thaliana*	SCAB1	Remodeling the actin cytoskeleton	SCAB1 coordinates sequential Ca^2+^ and ABA signals during osmotic stress induced stomatal closure [3].
Myosin	*Arabidopsis thaliana*	AtXI-K, AtXI-2, AtXI-1	Actin organization	Myosin mutants (*atxi-k katxi-2 atxi-1*) increase sensitivity to drought stress [98].
	*Oryza sativa*	OsMYA1	Actin organization	The *OsMYA1* knockout mutant exhibited decreased resistance to M. oryzae infection [99].
LIM domain-containing protein	*Arabidopsis thaliana*	WLIM2A	Cytoskeleton organization	The *wlim2a* lines were compromised in their response to *Pseudomonas syringae* Pst DC3000 but showed enhanced resistance to the necrotrophic fungus *Botrytis cinereae* [100].
LIM domain-containing protein	*Triticum aestivum* L.	TaLIM	TaLIM8-4D	*TaLIM8-4D* is significantly induced by heat, drought, sodium chloride (NaCl), abscisic acid (ABA) and *Fusarium graminearum* stresses. And overexpression of TaLIM8-4D could upregulate plant pathogenesis-related (PR) genes, promoting the infection of hemibiotrophic pathogen [101].
Capping protein	*Arabidopsis thaliana*	AtCPB	Regulates assembly at the barbed ends of actin filaments	AtCPB negatively regulates thermotolerance in *Arabidopsis* [102]. And defense responses are impaired in the *cpb-1* mutant [103].
Actin-binding protein, ABP	*Arabidopsis thaliana*	PGSL1	Binds and stabilizes actin filaments	PGSL1 enhances pollen germination and tube growth at high temperature [104].

## Data Availability

No new data were created or analyzed in this study. Data sharing is not applicable to this article.
